# Toxoplasmosis meets the World Health Organization criteria for a neglected tropical disease

**DOI:** 10.1371/journal.pntd.0014425

**Published:** 2026-06-25

**Authors:** João Marcello Furtado, Rubens Belfort Jr, Bahram Bodaghi, Soon-Phaik Chee, Alejandra de-la-Torre, Oleksandra Dorokhova, Moncef Khairallah, Padmamalini Mahendradas, Nadine Nsiangani-Lusambo, Uwe Pleyer, Serge Resnikoff, Frank Seeber, Hugh R. Taylor, Jennifer E. Thorne, Daniel V. Vasconcelos-Santos, Steven Yeh, Justine R. Smith

**Affiliations:** 1 Division of Ophthalmology, Ribeirão Preto Medical School, University of São Paulo, Ribeirão Preto, Brazil; 2 Department of Ophthalmology, Federal University of São Paulo, São Paulo, SP, Brazil; 3 Department of Ophthalmology, Pitié-Salpêtrière University Hospital, Sorbonne University, Paris, France; 4 Department of Ophthalmology, National University Hospital, Singapore, Singapore; 5 Neuroscience Research Group, Neurovitae Center for Neuroscience, Institute of Translational Medicine, School of Medicine and Health Sciences, Universidad del Rosario, Bogotá, Colombia; 6 The Filatov Institute of Eye Diseases and Tissue Therapy of NAMS of Ukraine, Odesa, Ukraine; 7 Department of Ophthalmology, Fattouma Bourguiba University Hospital, Monastir, Tunisia; 8 Department of Uveitis and Ocular Immunology Services, Narayana Nethralaya, Bengaluru, India; 9 Department of Ophthalmology, Teaching Hospital of Kinshasa, University of Kinshasa, Kinshasa, Democratic Republic of Congo; 10 Department of Ophthalmology, Charité-Universitätsmedizin Berlin, Berlin, Germany; 11 Brien Holden Vision Institute, University of New South Wales, Sydney, Australia; 12 Unit 16: Mycotic and Parasitic Agents and Mycobacteria, Robert Koch-Institute, Berlin, Germany; 13 Minum Barreng: Indigenous Eye Health Unit, Melbourne School of Population and Global Health, University of Melbourne, Melbourne, Australia; 14 Wilmer Eye Institute, The Johns Hopkins University School of Medicine, Baltimore, United States of America; 15 Department of Ophthalmology and Otorhinolaryngology, Faculty of Medicine, Federal University of Minas Gerais, Belo Horizonte, Minas Gerais, Brazil; 16 Ophthalmology and Visual Sciences, University of Nebraska Medical Centre College of Medicine, Omaha, United States of America; 17 Flinders Health and Medical Research Institute, and College of Medicine and Public Health, Flinders University, Adelaide, Australia; Xuzhou Medical University, CHINA

*Toxoplasma gondii* is a protozoan parasite that is transmitted to humans primarily by ingestion of raw or undercooked meat containing tissue cysts, or fresh produce and water contaminated with oocysts shed by the definitive feline host [1]. While less common, the heaviest health burden arises when the parasite is transmitted across the placenta of a newly infected pregnant woman to her fetus. In this context, a typically subclinical infection in the mother may be the cause of miscarriage, or lifelong neurological and/or visual impairment [1].

Toxoplasmosis continues to be one of the most common parasitic infectious diseases affecting humans, and the leading intraocular infection worldwide [1]. Yet, the condition receives limited attention on health agendas. Despite a global distribution, its most severe consequences disproportionately affect populations already facing social exclusion, restricted access to clinical services, and multiple health vulnerabilities [2]. Although toxoplasmosis is often regarded as a zoonosis that is an unavoidable consequence of everyday human–animal interactions [1], accumulated evidence indicates otherwise: toxoplasmosis has well-characterized pathways of transmission, and is preventable and controllable. This Viewpoint argues that toxoplasmosis fully meets all criteria set down by the World Health Organization (WHO) for recognition as a neglected tropical disease (NTD) [3] on public health and policy grounds, regardless of the taxonomic grouping of *T. gondii*. Importantly, official recognition as an NTD could advance research, capacity building, and public health action to address the substantial burden of toxoplasmosis.

## Toxoplasmosis is associated with poverty, especially in tropical and subtropical regions

A core criterion for NTD designation by the WHO is a disproportionate burden among populations living in poverty [4]. Toxoplasmosis clearly meets this marker. Studies from Latin America, the Middle East, and Africa consistently report higher infection risks in households with limited access to safe water, inadequate sanitation, variable food safety conditions, and substandard health care [5]. Congenital toxoplasmosis shows particularly stark social gradients. In low-resource settings, restricted access to antenatal care equates with missed opportunities for early diagnosis, counseling, and treatment. Infants who survive congenital infection face lifelong health consequences, such as visual and neurologic impairments, which undermine school performance, employment opportunities, and economic participation. These negative outcomes contribute to the poverty trap that exists for other NTDs: families already facing socioeconomic vulnerability absorb the long-term costs of disability, repeated medical visits, and lost income.

Toxoplasmosis is relatively prevalent in tropical and subtropical regions. Environmental factors typical of these climates, such as warm temperatures, humidity, and high rainfall, promote oocyst survival, and thereby, contamination of soil, surface water, and produce. Regions such as South America, where highly virulent genotypes circulate, exhibit some of the highest rates of ocular disease worldwide [6]. This geographical concentration of the disease aligns toxoplasmosis with established NTDs whose epidemiology is shaped by climatic and environmental determinants.

## Toxoplasmosis is amenable to prevention and control

Although prevention or control of a disease is inherently less attractive to WHO Member States than elimination or eradication, this should not obscure the fact that toxoplasmosis is preventable through measures already embedded in routine public health practice. Unlike multiple recognized NTDs, control would not hinge on a single intervention, such as mass drug administration. Instead, progress would be made by reinforcing systems to which these countries have already committed: stronger primary-care platforms capable of identifying maternal seroconversion during pregnancy; more consistent provision of prenatal care; improved sanitation and management of environmental contamination; and safer food production and handling. A combination of these measures may have contributed to the apparent decline in congenital toxoplasmosis in high-income countries [7]. These components are closely aligned with current WHO policies [8], including strengthening of primary health management, integrated people-centered care, and multisectoral actions on water, food safety, and environmental health. In this sense, even if toxoplasmosis lacks the single “hero intervention” that has characterized prior NTD successes, it fits naturally within the existing NTD portfolio of the WHO.

## Toxoplasmosis is underrecognized in global health research agendas

Consistency with the final WHO criterion for an NTD—relative neglect in research and development—is evident. Drawing on data provided by the United States National Institutes of Health, which is the largest global research funding agency, toxoplasmosis research was funded at a level of $177 per disability-adjusted life-year (DALY) for the period 2018–2024, compared with research on trachoma and Chagas disease, at $283/DALY and $337/DALY, respectively. Key gaps persist across basic science, diagnostics, therapeutics, prevention, and implementation research. No licensed human vaccine exists. Serological testing is widely available, but expensive for low-income scenarios and poorly standardized, complicating surveillance and estimation of the burden of disease. Treatment protocols lack robust comparative evidence, particularly for congenital and ocular toxoplasmosis. Environmental monitoring of oocysts remains technically demanding and absent from national programs.

This research deficit has direct consequences. Global disease burden estimates remain uncertain, and some regions with high prevalence lack up-to-date statistics. Without a formal designation as an NTD, toxoplasmosis competes unsuccessfully for resources, and it remains outside coordinated global strategies and is frequently absent from national health plans. The exclusion only reinforces ongoing misperceptions that toxoplasmosis is rare, or benign, or too complex to address.

Despite the uncertainties, available estimates still indicate a considerable burden, with roughly 190,000 congenital infections each year [2] and about 1.68 million DALYs [9], primarily due to the congenital infections, which rank among the highest DALYs of all foodborne diseases. The burden is not evenly distributed: South America carries the highest level, with additional concentrations in parts of the Middle East and multiple low-income countries [2].

## Why official recognition matters

Designation of toxoplasmosis as an NTD would confer practical benefits. First, it would secure visibility within the WHO global NTD framework, ensuring that toxoplasmosis is incorporated into monitoring systems, country reporting, and the Sustainable Development Goals 3.3 indicator of populations requiring NTD interventions [10]. Second, it would unlock funding streams dedicated to NTD research and the implementation of toxoplasmosis prevention and control measures, stimulating development of improved diagnostics, prenatal interventions, environmental surveillance tools, and meat-safety measures. Third, it would facilitate technical guidance for Ministries of Health, helping Member States integrate toxoplasmosis into mother–child health programs, food safety systems, and primary-care protocols. Finally, recognition would place toxoplasmosis within the global One Health agenda, enabling coordinated planning across the human health, veterinary, agricultural, and environmental sectors internationally [11,12].

These benefits are not symbolic. Other diseases have experienced immediate gains after receiving NTD designation: the adoption of integrated action plans, the emergence of new diagnostic and treatment pipelines, and measurable reductions in disease burden. Given the congenital consequences, including loss of the unborn child, and the substantial contribution to childhood and adult vision impairment and blindness [12], there is no reason toxoplasmosis should remain excluded from the group. Beyond terminology, continued exclusion of toxoplasmosis from the WHO NTD framework is likely to perpetuate weak visibility, fragmented surveillance, limited research investment, and insufficient policy coordination. This translates to missed opportunities to strengthen antenatal screening, improve food and water safety, expand access to timely diagnosis and treatment, and develop integrated One Health approaches. The result is not merely institutional delay, but new preventable congenital infections, vision loss, and long-term disability, especially among populations already affected by poverty and limited access to care.

## A roadmap for coordinated action

A pragmatic roadmap for toxoplasmosis would build on existing WHO NTD pillars:

- **Innovative and intensified disease management:** standardized algorithms for prenatal screening, diagnosis, and treatment of congenital infections; clinical guidelines for management of ocular toxoplasmosis; improved access to essential medicines and rehabilitation services for those with visual or neurological impairment; curative drugs and an effective vaccine for use in humans.
- **Preventive measures:** strengthened food safety policies for meat, water, and produce; community-level education; environmental measures to reduce human exposure to oocysts.
- **Transmission control:** integration of veterinary public health strategies, including improved farm biosecurity, management of stray cats, and safe disposal of animal waste.
- **Capacity building:** training mother–child health professionals, eye care professionals, laboratorians, veterinarians, and environmental scientists; incorporating toxoplasmosis into primary-care curricula.
- **Operational research:** dissemination of accurate and affordable diagnostics; evaluation of cost-effective screening strategies; burden-of-disease studies in underrepresented regions; testing of new tools to detect and monitor environmental contamination.

Coordinated cross-sectoral collaboration should be pursued over parallel management structures. Mother–child health services can support prenatal screening, counseling, and treatment; primary care can reinforce prevention messages and referral pathways; veterinary and agricultural sectors can contribute to farm biosecurity, management of livestock, and the safe disposal of animal waste; food safety authorities can strengthen measures related to handling of meat and fresh produce, and water safety; environmental agencies can help reduce contaminations relevant to human exposure. More broadly, the experience gained from established NTD programs suggests that sustained progress is most likely when control efforts are integrated into existing multisectoral systems rather than being pursued through isolated, disease-specific structures.

This roadmap would not, and cannot, aim at eradication of toxoplasmosis, but at measurable reductions in foodborne and environmental transmission, congenital infections, and ocular disease. These objectives are realistic, align with existing infrastructures in many countries, and would yield major long-term social and economic gains.

## Final comments

We conclude that toxoplasmosis qualifies for classification as an NTD by the WHO: it more often affects populations facing poverty, especially in tropical and subtropical regions; it can be prevented or treated through public health interventions; and it receives less attention and research funding than less-impactful diseases (Table 1). Without this recognition, we can expect limited progress in the prevention and management of toxoplasmosis to continue, despite the availability of practical interventions to address preventable disability and death. Adding toxoplasmosis to the NTD portfolio is not without risks: this action could strain resources that are already limited and dilute the efforts underway in existing programs for other NTDs. However, given the DALYs associated with toxoplasmosis, the benefits of proceeding far outweigh these risks; moreover, those risks could be mitigated by a proportional investment and clear strategic prioritizations. Recognizing toxoplasmosis as an NTD would align global policy with disease epidemiology and enable the innovations and partnerships that are required to reduce its burden. At a time when the WHO NTD framework emphasizes equity, integration, and multisectoral action, toxoplasmosis represents a clear and actionable gap that warrants corrective action. Our Viewpoint is a call to action to finally address the unacceptable global health burden of toxoplasmosis.

**Table 1:** Toxoplasmosis viewed against the World Health Organization (WHO) criteria for Neglected Tropical Disease (NTD) designation and compared with examples of designated NTDs.

WHO criterion for NTD designation	Relevant features	Comparison with designated NTDs
Burden concentrated in populations affected by poverty	Higher risk is observed where access to safe water, sanitation, food safety, and prenatal care is limited. The most severe consequences, particularly congenital infection and vision-threatening ocular disease, disproportionately affect socially vulnerable populations and may reinforce social and economic disadvantage long-term.	As with trachoma and Chagas disease, the burden is shaped not only by biological exposure, but also by poverty-related barriers to prevention, early diagnosis, treatment, and long-term care.
Important burden in tropical and subtropical settings	Environmental conditions in tropical and subtropical regions favor oocyst survival and transmission. In parts of South America, severe ocular disease is particularly frequent, perhaps reflecting the circulation of more virulent *Toxoplasma gondii* strains, together with contextual social and environmental factors.	Similar to leishmaniasis and schistosomiasis, the epidemiology and burden of toxoplasmosis are strongly influenced by climatic and environmental conditions that are common in tropical and subtropical settings.
Amenable to prevention and control	Prevention and control can be strengthened through improved antenatal care, earlier diagnosis and treatment, safer food preparation, better water and sanitation, reduction of environmental contamination, and coordinated veterinary and environmental actions within a One Health framework.	Like dengue and snakebite envenoming, toxoplasmosis is compatible with a public health approach centered on reducing exposure, morbidity, and long-term consequences, rather than eradication.
Neglected in research and policy	Research investment, surveillance, and technical guidance remain insufficient relative to disease burden. Important research gaps persist in diagnostics, treatment optimization, prevention strategies, burden estimation, and integration into national and international health agendas.	Toxoplasmosis presents a similar scenario to Chagas disease and trachoma, but has received substantially lower research funding per DALY from the United States National Institutes of Health.
